# Genome Engineering in *Vibrio cholerae*: A Feasible Approach to Address Biological Issues

**DOI:** 10.1371/journal.pgen.1002472

**Published:** 2012-01-12

**Authors:** Marie-Eve Val, Ole Skovgaard, Magaly Ducos-Galand, Michael J. Bland, Didier Mazel

**Affiliations:** 1 Institut Pasteur, Unité Plasticité du Génome Bactérien, Département Génomes et Génétique, Paris, France; 2 CNRS, URA2171, Paris, France; 3 Department of Science, Systems, and Models, Roskilde University, Roskilde, Denmark; University of Toronto, Canada

## Abstract

Although bacteria with multipartite genomes are prevalent, our knowledge of the mechanisms maintaining their genome is very limited, and much remains to be learned about the structural and functional interrelationships of multiple chromosomes. Owing to its bi-chromosomal genome architecture and its importance in public health, *Vibrio cholerae*, the causative agent of cholera, has become a preferred model to study bacteria with multipartite genomes. However, most *in vivo* studies in *V. cholerae* have been hampered by its genome architecture, as it is difficult to give phenotypes to a specific chromosome. This difficulty was surmounted using a unique and powerful strategy based on massive rearrangement of prokaryotic genomes. We developed a site-specific recombination-based engineering tool, which allows targeted, oriented, and reciprocal DNA exchanges. Using this genetic tool, we obtained a panel of *V. cholerae* mutants with various genome configurations: one with a single chromosome, one with two chromosomes of equal size, and one with both chromosomes controlled by identical origins. We used these synthetic strains to address several biological questions—the specific case of the essentiality of Dam methylation in *V. cholerae* and the general question concerning bacteria carrying circular chromosomes—by looking at the effect of chromosome size on topological issues. In this article, we show that Dam, RctB, and ParA2/ParB2 are strictly essential for chrII origin maintenance, and we formally demonstrate that the formation of chromosome dimers increases exponentially with chromosome size.

## Author Summary

*Vibrio cholerae*, the causative agent of cholera in humans, has two circular chromosomes of uneven size, each with distinct maintenance requirements. This is in contrast to classical, *Escherichia coli*–centric bacterial models of a single chromosome. In this study, we took advantage of *V. cholerae*'s atypical genome structure to address important biological issues related to the maintenance of multipartite genomes. We further used *V. cholerae* to determine how genome architecture and genetic organization affects the odds of topological difficulties arising during replication. Our approach consisted of performing massive genome rearrangements to create various synthetic mutants of *V. cholerae* with nearly identical genetic backgrounds. We created mutants of *V. cholerae* with a single chromosome, with two chromosomes of equal size, or with identical origins of replication. To do so, we developed a genetic engineering tool based on the multiplexing of two site-specific recombination systems to allow efficient and directional manipulations of any DNA segment. In this study, we show that Dam, RctB, and ParA2/ParB2 are only essential for chrII origin maintenance, and we demonstrate that the odds of forming chromosome dimers exponentially increases with chromosome size.

## Introduction

Bacteria were long thought to have a simple genome architecture based on a unique circular chromosome, and it is only in the late 1980s that the first prokaryote with multiple chromosomes, *Rhodobacter sphaeroides*, was characterized [1]. Since this seminal observation, many other species possessing multiple circular or linear chromosomes have been characterized across numerous bacterial lineages [2]. More than 80 multipartite bacterial genomes have been sequenced, propagating various hypotheses to explain their extant nature and posing fundamental questions about the selective benefit of such a genome architecture.

Numerous studies have established the cholera pathogen, *Vibrio cholerae*, as the model for bacteria with multipartite genomes [3]. The genome of *V. cholerae* N16961 consists of two circular chromosomes, a primary 2.96 Mbp chromosome (chrI) and a secondary 1.07 Mbp chromosome (chrII). *V. cholerae's* genes are asymmetrically distributed between the two chromosomes [4]. ChrI has low interspecies sequence variability and harbors many genes coding for essential biosynthetic pathways. ChrII contains many more species-specific genes, unknown ORFs and proportionally fewer essential genes [4]–[5]. Furthermore, *V. cholerae*'s particular genomic organization and genetic disparity is consistent within the *Vibrionaceae* family [6]–[8]. The unusual genome structure of *V. cholerae* has inspired numerous studies to better understand the mechanisms and purposes of maintaining such a genomic organization, resulting in an impressive body of experimental data [9]–[20]. To date, however, and despite the impressive collective effort of the cited studies along with other research on chromosome and plasmid maintenance systems, the mechanisms coordinating the maintenance of multiple chromosomes are largely unknown. In tackling such pervasive yet fundamental questions, we decided to construct a unique genetic tool allowing targeted massive chromosomal rearrangements in proteobacteria. We applied this powerful technique to answer two outstanding questions. Firstly, we addressed the specific case of the essentiality of Dam methylation in *V. cholerae*. Secondly, we focused our genetic system on more general questions concerning bacteria with circular chromosomes by examining the effect of chromosome size and genetic distribution on topological issues.

Unlike eukaryotic organisms, where chromosomes are managed by common machineries which coordinate up to 90 chromosomes [21], *V. cholerae* has evolved a relatively complex and highly targeted strategy involving interplay of specific and common machineries for the maintenance of each chromosome. Replication of each *V. cholerae* chromosome is controlled by a unique initiator molecule [11]. ChrI replication is initiated at *oriI* by DnaA, the common initiator of chromosomal DNA replication in most bacteria [11], while chrII replication is regulated at a plasmid-like *oriII* by the *Vibrio*-specific factor, RctB [22]–[23]. ChrII is nonetheless replicated only once per cell generation, unlike plasmids, which are not generally linked to the cell cycle [24]. Both *E. coli* and *V. cholerae* are members of a mono-phyletic clade of the gamma-proteobacteria defined by the acquisition of the dam-seqA-mutH genes ensuring restriction of chromosome replication initiation to once per cell cycle and probe mismatch repair of replication errors [25]. Dam methylates the palindromic GATC sequence on both strands, which become transiently hemi-methylated after replication. The origin of replication and other regions with clusters of GATC sites become sequestered after replication by SeqA for up to one third of the cell cycle, which serves to preclude new initiations of replication [25]. Both *V. cholerae* chrI and chrII origins have GATC methylation sites [12] and their sequestration by SeqA contributes to limiting initiation of DNA replication to only once per cell cycle [9]. Replication of the larger chrI is initiated significantly before chrII so as to insure replication is terminated synchronously, suggesting a coordinating mechanism which has yet to be explained [16]. Whereas Dam is not an essential factor in *E. coli*, *V. cholerae* mutants lacking Dam methylation are not viable [26], implying the existence of differences in replication regulation between the two organisms. Methylation by CcrM, the counterpart of Dam in the α-proteobacteria, is also known to be essential for the viability of bacteria with multipartite genomes [27]–[29]. For these reasons, it was strongly suspected that the crucial role of Dam in *V. cholerae* could be related to its atypical genome arrangement, and Dam appeared to be a good candidate to investigate the coordinated replication initiation of the two chromosomes. *In vitro* studies showed that Dam methylation of RctB binding sites increases RctB binding and possibly serves a critical function in chrII replication [9]. The requirement for Dam in order to initiate replication at *oriI* was first studied *in vivo* using plasmids and monitoring the transformation efficiency of plasmids driven by *oriI*. These plasmids failed to transform *E. coli dam* mutants suggesting that Dam was essential for *oriI* replication initiation [12]. A reciprocal experiment involving *oriC*-plasmids and a mutant of *E. coli* where *oriC* was substituted by *V. cholerae oriI* (*ΔoriC::oriI*) showed that *oriC*-plasmids failed to transform *E. coli ΔoriC::oriI Δdam* [9]. Confronted with this last result and knowing that Dam is not essential in *E. coli*, it was hypothesized that the additional *oriI*-plasmid copies out-competed replication from chromosomal *oriC*, thus creating incompatibility conditions where Dam was required for viability of the transformants [9]. To prevent plasmid-mediated competition, the Dam requirement of *V. cholerae oriI* was directly assessed on the chromosome in *E. coli ΔoriC::oriI* [9], [30]. Two conflicting experiments, differing from the manner in which *oriC* was substituted by *oriI*, showed Dam methylation to be either required for the viability of *E. coli ΔoriC::oriI* [30] or not [9]. Therefore, the question of whether Dam was essential or dispensable for replication initiation of *V. cholerae oriI* remained unresolved. Reciprocal experiments consisting of testing *oriII* Dam requirement in a *E. coli* chromosomal context could not be tested because all attempts to replace *oriC* with *oriII* were unsuccessful [9].

After replication, partitioning of the resulting homologous chromosomes is fundamental to maintain genome stability [31]. In *V. cholerae*, the segregation of *oriI* and *oriII* are mediated by distinct partition factors, ParA1/B1 for chrI and ParA2/B2 for chrII [13]. ParA/B partitioning activity requires centromere-like, *cis*-acting sites called *parS*, which are bound by ParB to form a nucleoprotein complex that is a target for the ParA ATPase protein [32]. ParA1/B1 are chromosomal-like and mediate an asymmetric segregation of *oriI* [14]. On the other hand, ParA2/B2 are plasmid-like and carry out a symmetric segregation of *oriII* [20]. ParA1/B1 are not essential for chrI segregation, indicating that other factors contribute to the segregation of chrI [14] while ParA2/B2 are essential for chrII segregation and cell viability [20]. Many other bacteria with multipartite genomes have integrated distinct plasmid-like origins of replication and partitioning mechanisms to maintain their secondary chromosomes [3], which supports the hypothesis that secondary chromosomes were originally acquired as megaplasmids.

*V. cholerae* uses an interesting combination of mechanisms derived from both chromosomes and plasmids for the maintenance of chrII. In contrast to the above-mentioned plasmid-like mechanisms, terminal segregation of both chrI and chrII is controlled by a common *bona fide* chromosomal maintenance system involved in the generation of monomeric chromosome substrates for partitioning [19]. Circular chromosomes convey specific topological problems, such as the formation of dimeric chromosomes, which threatens the partition of genetic information to daughter cells (for a review, see [33]). Chromosome dimers are a side-product of homologous recombination associated with recombinational DNA repair between replicating or newly replicated circular chromosomes [33]. If an odd number of crossovers occur between sister strands, chromosome dimers are formed and must be resolved into monomers to allow chromosome segregation. This process is carried out by the combined action of the site specific tyrosine recombinases XerC and XerD that introduce an additional crossover at *dif*, a 28 bp site located opposite of the origin of replication [33]. *V. cholerae* carries two distinct recombination sites, *dif1* and *dif2*, located in the terminus region of chrI and chrII, respectively [19]. Resolution of chromosome dimers of chrI and chrII links chromosome segregation to the late stages of cell division via the septal protein FtsK [19].

The presence of multiple chromosomes has posed challenges for *in vivo* studies of chromosome maintenance in bacteria as it is difficult to attribute observed phenotypes to a specific chromosome. To circumvent this issue, we designed a strategy based on specific genome rearrangements to directly study biological systems in their endogenous host. We developed a genetic tool based on two distinct site-specific recombination machineries, which allow targeted, oriented and reciprocal DNA exchanges throughout the genome. We used *V. cholerae* as a bi-chromosomal bacterial model to show the power of our genetic tool and how its use can help address important biological questions. Using this strategy, we examined the requirement of *Vibrio*-specific essential factors involved in chromosome maintenance for which functions could not be strictly attributed to a specific chromosome. We also investigated the correlation between chromosome size and the rate of formation of chromosome dimers that are the inevitable by-products of frequent recombination associated with recombinational DNA repair. To address all these questions, we created a mutant of *V. cholerae* with all its genetic content reorganized onto a single chromosome. We further refined our study by making additional chromosomal rearrangements to individually decipher each biological issue. In this article, we show that Dam, RctB and ParA2/ParB2 are only essential for chrII origin maintenance. We further demonstrate that the odds of forming chromosome dimers exponentially increases with chromosome size.

## Results/Discussion

### One from two: Reorganizing the genome of *V. cholerae*

We generated a mutant of *V. cholerae* with all its genetic content reorganized onto a single chromosome. To do so, we fused chrI with chrII in a calculated and conservative manner respecting known criteria for chromosome organization and maintenance. Prokaryotic genomes show intolerance towards various chromosome rearrangements such as inversions or relocations of DNA fragments [34]–[44]. Nevertheless, bacterial chromosomal structure can be drastically altered [45]–[48] provided that organizational features are respected (for reviews [49]–[51]). The fused chromosome was constructed to conserve the “*ori*-*ter*” axial symmetry, gene synteny, strand bias and the polarities of the original replichores. Replication of the fused chromosome initiates at *oriI* of chrI and finishes in the terminus of chrII near *dif2*. The single fused chromosome carries exclusively chromosomal-like attributes for replication and chromosome segregation (*oriI*, *ParA1/B1*, *dif2*), like other mono-chromosomal bacteria. By initiating replication at *oriI*, we conserve the replication-associated gene dosage on chrI [10]. Lastly, comparative genomics has shown that the *ter* region of chrI is flexible and would likely tolerate the integration of the 1 Mbp chrII [7], [52].

To perform the above-mentioned genome rearrangements, we developed a genetic tool which allows efficient and directional manipulations of any DNA segment. It involves two site-specific recombination systems which normally promote precise excision of the temperate phage genomes, λ and HK022, from their chromosomal location [53]. We used λ and HK022 integrases (Int_λ_ and Int_HK_), their respective excision factors (Xis_λ_ and Xis_HK_) and their associated left and right excision sites (*attR_λ_/attL_λ_* and *attR_HK_/attL_HK_*). Unlike other site-specific recombination systems used for precise genome manipulation such as Cre/*loxP* [54] or Flp/*FRT* [55], the λ and HK022 recombination reactions have the calculated advantage of being directionally controlled, as the presence of the Xis excision factors orientates the catalytic reactions in one direction. This characteristic is very useful for two reasons: first, it insures that the mutant strain will not revert to the wild-type configuration after chromosomal rearrangement. Second, the newly formed sites (*att*B/P) react poorly with the substrate sites (*att*R/L) [56]. Therefore the same system can be reused in the mutant strain to perform new rearrangements at other positions by integrating new *att*R/L sites. In theory, this system could be used an infinite number of times in the same strain.

**Figure 1:**
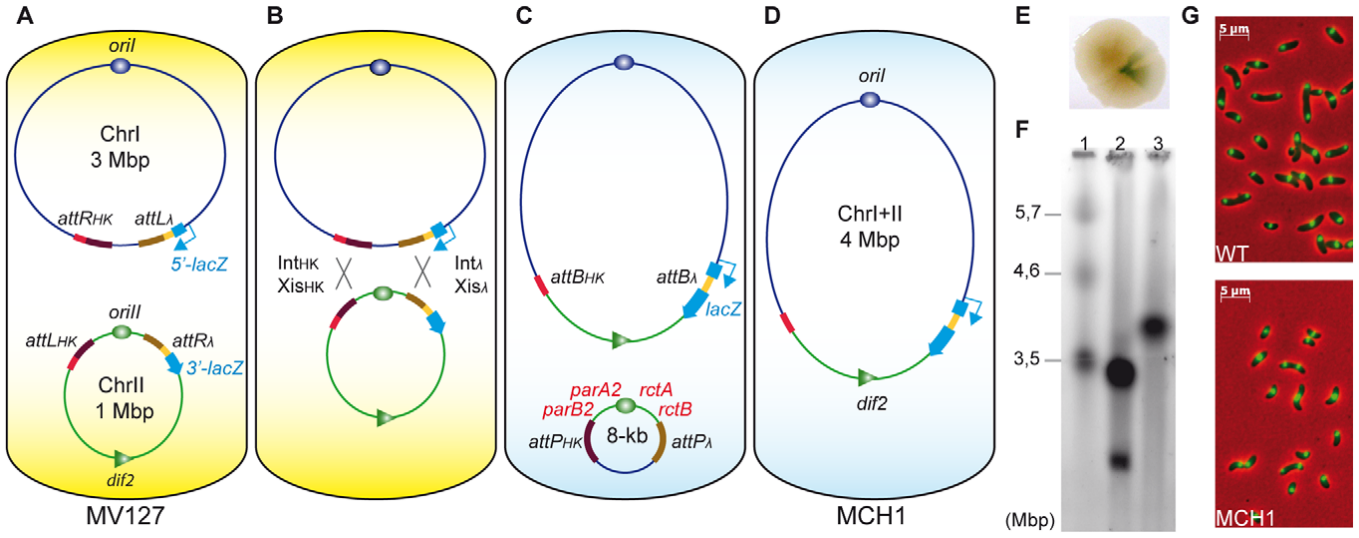
A mono-chromosomal *V. cholerae* model, MCH1. A. *V. cholerae* MV127 strain with *attR/L* sites from λ and HK022 phages inserted at precise loci. Recombination sites [*attR_HK_* and *attL_λ_*] replaced *dif1* on chrI and [*attL_HK_* and *attR_λ_*] flanked [*parAB2-oriII-rctAB*] on chrII. B. Recombination [*attR_λ_*×*attL_λ_*] and [*attR_HK_*×*attL_HK_*] mediated by the expression of Int_λ_+Xis_λ_ and Int_HK_+Xis_HK_. C. Recombination events [*attR_λ_*×*attL_λ_*] regenerate *lacZ*, allowing for phenotypic detection of rearranged chromosomes. D. Without selection, the 8 kb excised molecule (carrying a kanamycin resistance gene) was lost. E. Blue sector appearing within single conjugant on X-Gal supplemented LB-agar plates indicates recombination events between [*attR_λ_*×*attL_λ_*]. F. Ethidium bromide stained PFGE of genomic DNA: Lane 1, *S. pombe* marker (BioRad) ; Lane 2, WT (N16961) ; Lane 3, MCH1. G. Microscopic observation of WT (top panel) versus MCH1 (bottom panel). Nucleoids of exponentially growing cells stained with DAPI (green) merged with phase-contrast images (red).

To fuse the two chromosomes of *V. cholerae*, each partner a*ttL* and *attR* sites specific to the same integrase were inserted on separate chromosomes: *attR_HK_/attL_λ_* were inserted at the junction between the two replichores in the terminus region of chrI and *attL_HK_/attR_λ_* were placed flanking [*parA2/B2-oriII-rctA/B*] in the origin region of chrII (Figure 1A). The consecutive recombination reactions between *attR_HK_/attL_HK_* and *attR_λ_/attL_λ_* sites, upon expression of Int and Xis, led to the fusion of chrI with chrII (Figure 1B, 1C). To visualize chromosomal rearrangement events, we used a colorimetric screen based on recombination-dependent reconstitution and expression of the *lacZ* gene (Figure 1E). We obtained a stable MonoCHromosomal *V. cholerae* mutant strain (MCH1) with a single chromosome of the expected 4 Mbp size (Figure 1F) observable by pulsed field gel electrophoresis (PFGE). MCH1 cells attain a generation time of 29 minutes when grown in fast-growing conditions (Table S1). Under the microscope, MCH1 fixed cells are indistinguishable from N16961 wild-type (WT) (Figure 1G) and the counting of viable cells forming microcolonies confirmed that MCH1 incurs no increase in the rate of mortality compared to the WT (data not shown). We measured the DNA distribution in exponentially growing cultures by flow cytometry and compared these distributions with modeled distributions (Figure S1). Whereas WT has a replication pattern which can be successfully modeled by assuming that chrII initiates late and terminates at approximately the same time as chrI as previously described [16], our analysis of MCH1's replication pattern was consistent with a single chromosome replicated at a constant rate (Figure S1).

### RctB initiator and ParA2/B2 partitioning factors are essential for chrII maintenance only

We have taken a radical genetic approach by rearranging the genome of *V. cholerae* to investigate the specific biological functions of RctB and ParA2/B2. Since chrII is indispensable, these factors, essential for chrII initiation and partition, are ultimately essential for cell viability [11], [20], [22]. However, an additional role in the maintenance of chrI could never be formally tested due to the essentiality of their functions for chrII perpetuation. Recombinational fusion of the two chromosomes in MCH1 resulted in the excision of an 8 kb circular molecule carrying [*parA2/B2*-*oriII*-*rctA/B-aph*] (Figure 1C). The excised molecule encoded a functional *aph* gene conferring kanamycin resistance to the parental strain of MCH1, MV127. This circular molecule was readily lost in absence of selection observable by the absence of kanamycin resistance in MCH1 cells (Figure 1D). Loss of the 8 kb molecule was further confirmed by PCR, showing an absence of amplification of *parB2* and *rctB* loci from MCH1 genomic DNA, while these loci could normally be amplified from MV127 genomic DNA (data not shown). Loss of the 8 kb molecule was surprising since it harbored the *oriII* origin of replication and a centromere-like *parS2-B* site (within *rctA*) [57] along with associated replication (*rctA/B*) and partitioning (*parA2/B2*) factors that should allow it to replicate autonomously in the cell. We have no experimental evidence that could explain this loss, but it could be the result of partition-mediated incompatibility [58] between *parS2* sites located on separate entities, the fused chromosome and the 8 kb circular molecule. Yet, by physically linking chrII to chrI in MCH1, we placed replication and partitioning of chrII under the control of chrI machinery rendering chrII factors for replication initiation (RctB) and partitioning (ParA2/B2) non-essential.

Most of the centromere-like *parS2* sites are located near *oriII*, ensuring its partition, but a functional *parS2* site, *parS2-1*, was found located near the chrI terminus [57]. Therefore, ParA2/B2 could have an important function for the segregation of the terminus region of chrI. Under the microscope, MCH1 cells are indistinguishable from WT (Figure 1G). Nucleoid staining with DAPI shows no evident segregation or division problems that would be easily detectable by the presence of anucleoid cells, filaments and chromosomes trapped in the septum of division (Figure 1G). Our approach allowed us to readily demonstrate that the essential functions of RctB and ParA2/B2 in *V. cholerae* are strictly limited to chrII maintenance.

### The essential activity of Dam is restricted to replication initiation at *oriII*

**Figure 2:**
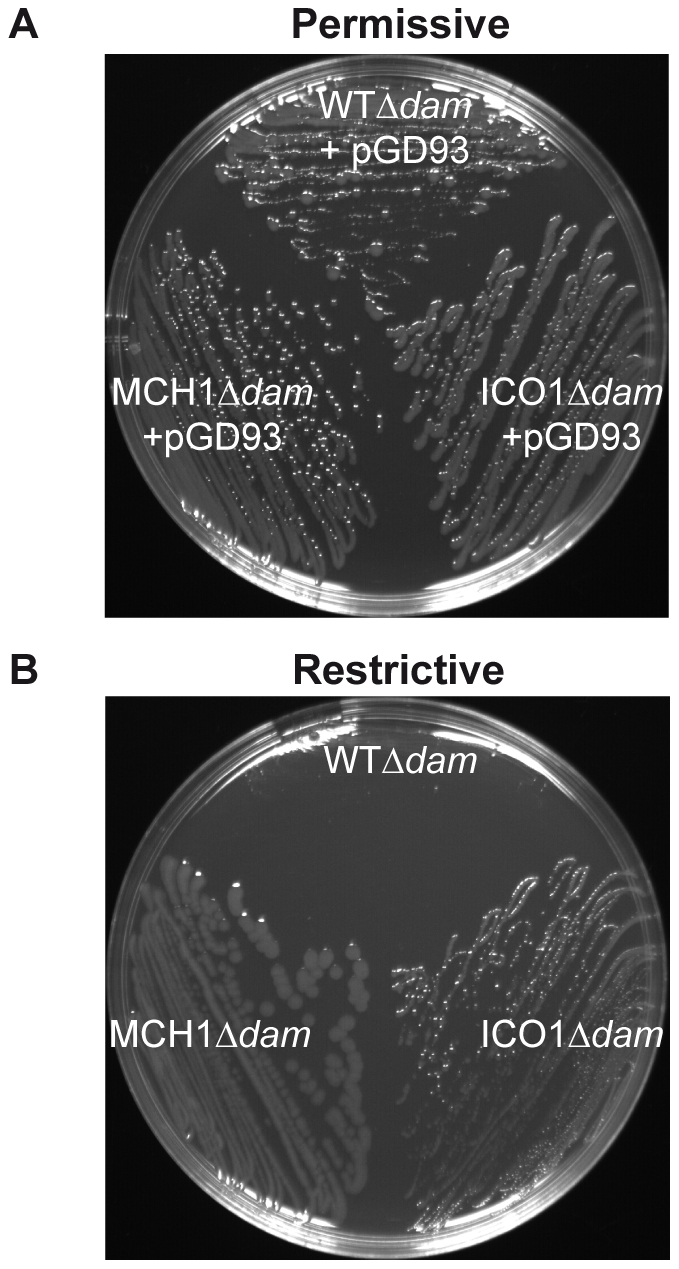
Dam is only essential for replication initiation of chrII from *oriII*. Growth of WTΔ*dam*/pGD93, MCH1Δ*dam*/pGD93 and ICO1Δ*dam*/pGD93 on LB-agar plates under (A) permissive conditions (+0.2% arabinose at 30°C, allowing *dam* expression and pGD93 replication) or (B) restrictive conditions (+1% glucose at 42°C, repressing *dam* and preventing pGD93 replication).

All previous *in vivo* Dam studies were undertaken in *E. coli*, where Dam is not essential. Here we investigate the essential function of Dam directly in *V. cholerae* to eliminate confusion arising from extrapolated results from *E. coli*. MCH1 enabled us to test the essentiality of Dam in replication initiation, since it only carries a single origin of replication, *oriI*. We deleted *dam* in MCH1 and the WT. Deletion of *dam* was done in the presence of pGD93, a complementing temperature sensitive replicating plasmid expressing *V. cholerae dam* under the control of an arabinose-inducible (permissive conditions) and glucose-repressible (restrictive conditions) promoter [9]. In the presence of Dam, both WTΔ*dam*-[pGD93] and MCH1Δ*dam*-[pGD93] grew normally (Figure 2A). Under restrictive conditions when Dam was depleted, WTΔ*dam* colonies were hardly visible (Figure 2B) confirming the essentiality of Dam in *V. cholerae*. MCH1Δ*dam*, on the other hand, grew and formed colonies under restrictive conditions (Figure 2B), indicating that Dam is no longer essential. This result demonstrates that initiation of replication at *oriI* doesn't require Dam. To more precisely characterize the role of Dam, we created a second mutant of *V. cholerae* where we maintained two distinct chromosomes but placed replication of chrII under the control of an additional copy of *oriI*, since Dam is not essential to initiate replication at *oriI*. To substitute *oriII* with *oriI*, we used the dual site-specific recombination tool previously described (Text S1). We generated a mutant of *V. cholerae* carrying two Identical Chromosomal-like *oriI* Origins (ICO1). The *rctB* deletion did not affect the viability of ICO1 confirming that its essential function was only required for replication initiation of *oriII*. We further tested the essentiality of Dam in ICO1, as described previously, and found that ICO1Δ*dam* cells were viable under restrictive conditions (Figure 2B) demonstrating that Dam is no longer essential when chrII replication is initiated at *oriI*. Therefore, we can assert that Dam is required for replication initiation of chrII from *oriII* only.

It was previously thought that the critical function of Dam in *V. cholerae* could be related to its atypical genome arrangement. However, Dam is also essential in *Yersinia pseudotuberculosis* and *Aeromonas hydrophila* [59], bacteria with single chromosomes and members of the gamma subdivision of proteobacteria with *V. cholerae*. Therefore, the essential function of Dam could possibly be unrelated to the management of a multipartite genome. It is known that DNA methylation exerts an effect on diverse bacteria via its role as a global regulator of gene expression. In *E. coli*, many genes involved in DNA mismatch repair, SOS response, motility, host-pathogen interactions or cell cycle regulation are mis-regulated in the absence of Dam [59]. Thus, the role of DNA methylation in diverse cellular processes via gene expression regulation could also explain Dam's essential function in *V. cholerae*. The viability of MCH1Δ*dam* mutants allowed us to rule out the potential role of Dam as an essential global regulator of gene expression since they have nearly the same genetic background as the WT where *dam* deletion is lethal. We further demonstrated in MCH1 that Dam was not required for initiation of replication at *oriI*. We tested the essentiality of Dam in the mutant ICO1, which carries two chromosomes both initiated at *oriI*. Since ICO1*Δdam* is viable, this precisely defined *oriII* as the region where Dam executes its essential function. This result substantiates earlier *in vitro* work showing that RctB preferentially binds methylated *oriII* [9]. We propose that in absence of Dam, GATC sites in *oriII* do not become methylated, preventing the binding of RctB to *oriII* and therefore precluding chrII replication initiation and maintenance which is fatal to the cell.

### Chromosome dimer formation increases exponentially with the size of the chromosome

**Figure 3:**
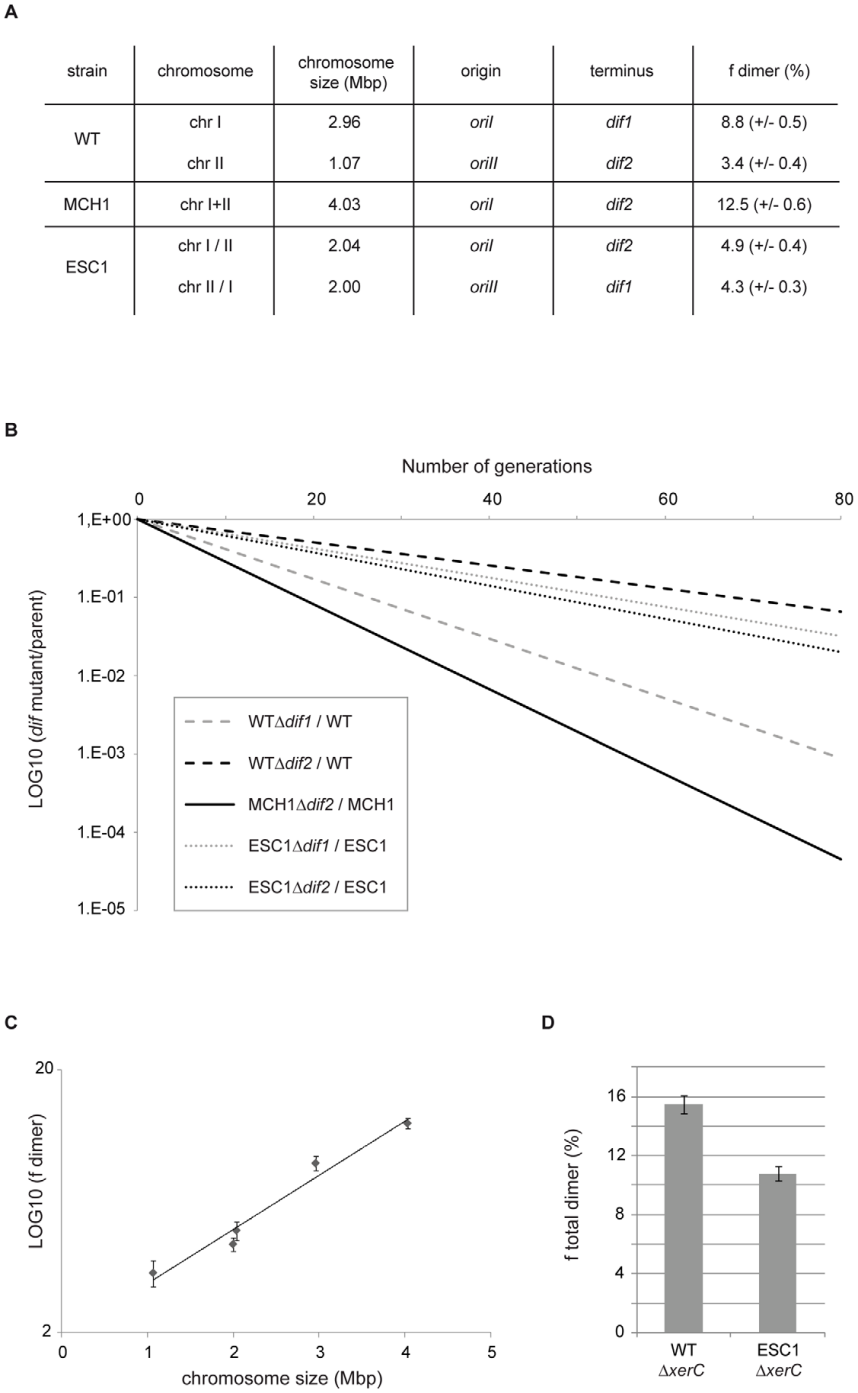
Chromosome dimer formation increases exponentially with the size of the chromosome. A. Table summarizing the chromosomal features of WT, MCH1 and ESC1. Frequency of dimers formed on each chromosome per cell per generation (f_dimer_) in percent (+/− standard error of the mean). B. Growth competition experiment to assay the activity of *dif* sites in chromosome dimer resolution. The logarithm of the ratio between the number of CFUs obtained with strains carrying *dif* sites and the number of CFUs obtained with their isogenic *Δdif* strains, is plotted as a function of the number of generations. Shown is the plot corresponding to the mean ratio of three independent experiments. C. Logarithm of the frequency of chromosome dimer formation plotted as a function of chromosome size. Dots display means of three independent experiments (+/− standard error of the mean). D. Histograms representing the total rate of chromosome dimers formed in WT and ESC1. Bar display the means of three independent experiments (+/− standard error of the mean) of the frequency of cells that the mutant strains (*ΔxerC*) failed to produce at each generation compared to their parents (in percent).

Formation of dimeric chromosomes is a particular problem associated with the circularity of bacterial chromosomes. We used *V. cholerae* as a bacterial model to determine how genome architecture affects the odds of topological difficulties during replication by assaying the effect of chromosome size on the rate of chromosome dimer formation. Very few cells carrying a dimer are expected to yield viable progeny in the absence of resolution. Inactivation of chromosome dimer resolution in *E. coli* results in ∼15% cell death per generation, which corresponds to the estimated rate of chromosome dimers formed at each cell generation [60]–[61]. We measured the fitness defect of a *dif* mutant by growth competition experiments, in which the growth of the mutant strain was directly compared to the growth of its parent (Figure 3B) to quantify the rate of dimers formed on a *dif*-carrying chromosome. In *V. cholerae* WT, 8.8% of dimers per cell per generation are formed on the 3 Mbp chrI (*Δdif1*) and 3.4% of dimers are formed on the 1 Mbp chrII (*Δdif2*) when grown in rich LB media (Figure 3A, 3B). In MCH1, 12.5% of dimers per cell per generation are formed on the 4 Mbp chromosome (Δ*dif*2) under the same growth conditions (Figure 3A, 3B). These results suggested that dimer formation increases with replicon size. To strengthen our interpretation, we decided to construct an additional mutant of *V. cholerae* with two equally sized chromosomes of 2 Mbp and measure the rate of dimer formation on each chromosome. We transferred 1 Mbp from chrI to chrII by swapping the 1.05 Mbp DNA fragment evenly surrounding *dif1* with the 0.12 Mbp DNA fragment evenly surrounding *dif2*, resulting in the exchange of *dif1* and *dif2* using the genetic tool described above (Text S1, Figure S2). We obtained a mutant of *V. cholerae* with Equally Sized Chromosomes (ESC1 with chrI/II and chrII/I) observable by PFGE (Figure S2D). A measure of the rate of chromosome dimers formed on the two 2 Mbp chromosomes was performed in ESC1. Our results show that 4.9% of dimers per cell per generation are formed on the 2 Mbp chrI/II (*Δdif2*) and 4.3% of dimers are formed on the 2 Mbp chrII/I (*Δdif1*) (Figure 3A, 3B). We plotted the rate of chromosome dimer formation as a function of chromosome size and observed a linear relationship between chromosome size and the logarithm of the frequency of dimer formation (r^2^ = 0.97) (Figure 3C, Methods). This result indicates that chromosome dimer formation increases exponentially with the size of the chromosome. In ESC1, the two equally sized chromosomes, chrI/II and chrII/I, have an asymmetric distribution of genes, specific machineries for their respective maintenance, distinct terminus regions and, very certainly, distinct chromosome structure, and yet the probability of dimer formation for each chromosome is essentially equivalent (Figure 3A). This implicated chromosome size as the primary influence on the rate of dimeric chromosome formation in an identical genetic background.

Homologous recombination involves a Holliday junction intermediate which is resolved by the RuvABC complex leading to either crossover or non-crossover potential products with only crossovers leading to the formation of chromosome dimers [62]. In *E. coli*, the RuvABC pathway is biased towards generating non-crossover products [63]–[64]. Since this bias can vary between species, it is not possible to infer the effects of genome architecture on the formation of chromosome dimers by direct comparison between bacteria with single and multiple chromosomes or between bacteria with multiple chromosomes of different sizes. *V. cholerae* allowed us to modify the size of the chromosomes by transferring DNA from one chromosome to the other, with minimal modifications of the genetic background.

### Genetic information distribution between the two chromosomes impacts chromosome dimer formation

We tested the effect of DNA distribution between multiple chromosomes on the total rate of chromosome dimer formation. To do so, we measured the fitness defect of *xerC* mutants to obtain a quantification of the total rate of chromosome dimers formed in the cells (Figure 3D). As a consequence of more dimer formation in WT compared to ESC1, we observed that a *xerC* deletion had a greater effect on WT than on ESC1 (15.5% in WT , 10.8% in ESC1). The unequal (WT) or equal (ESC1) genetic distribution influences the chances for chromosome dimers to arise. Based on this result, it might be considered surprising that the extant WT genome configuration has been selected and all vibrios characterized to date have been shown to possess two unequally sized chromosomes [8]. This suggests that dimer formation has little impact on the selection of DNA distribution on multiple chromosomes.

One possible explanation for *V. cholerae*'s unequally sized replicons and distinct replication initiation mechanisms may lie in adjusting the balance between genes found on separate chromosomes in response to drastic changes in growth conditions [10], [17], [65]. Gene dosage tends to shape chromosome organization of fast-growing bacteria, favoring placement of genes involved in translation and transcription near the origin of replication [65]. Differential gene dosage depends on replication rate, chromosome size and doubling time. This effect is particularly important for *V. cholerae* with its two chromosomes of uneven size and extremely short generation time. Indeed, when *V. cholerae* growth rate increases, origin-proximal loci of chrI are amplified by up to four copies per cell, yet origin-proximal loci of chrII never total more than two copies per cell [17]. Consistent with its larger size, gene dosage effects on chrI are greater than on chrII [10], [16]. Differently sized replicons may thus be selectively advantageous as a means to allow for a more nuanced gene dosage effect. This is certainly the case for the vibrios, where a higher abundance of growth-essential and growth-contributing genes are located near the origin of replication of chrI coupled with a dearth of such genes on chrII. This theory lends itself well to further investigation using our genetic engineering tools.

### New insights into bacterial genome organization

We developed a site-specific recombination-based engineering tool, which provides us with a powerful means to massively reorganize in principle any prokaryotic genome provided that necessary host factors are present. This genetic tool consists in harnessing the λ and HK022 recombination systems to perform a large panel of genome reorganizations. By controlling the location and the orientation of each partner recombination site, we can obtain a large variety of genome rearrangements, such as chromosome fusion (e.g. MCH1), transfer and exchange of DNA fragments (e.g. ESC1), deletion, insertion, inversion or substitution of DNA (e.g. ICO1). Thanks to the construction and analysis of various synthetic mutants, we were able to tackle important biological issues on chromosome maintenance in *V. cholerae*. We showed that Dam, RctB and ParA2/ParB2 factors are essential for chrII maintenance. We further revealed that the odds of forming chromosome dimers exponentially increase with the size of a circular chromosome.

Our construction of mutants with massive genome rearrangements demonstrates the incredible plasticity of prokaryotic genomes. All of these genomic mutants conserved the rapid growth characteristic of vibrios, although with a slightly extended generation time (Table S1) that may be linked to their alternative genomic structure. This is currently under investigation. Recent advancements in the field of synthetic biology have demonstrated that the *de novo* creation of artificial genomes is now an attainable objective [66]. The recent assembly of the 580 kb genome of *Mycoplasma genitalium* starting from chemically synthesized oligonucleotides [67] and the successful demonstration that one can maintain and engineer a bacterial genome in a yeast and then transfer it to a bacterial recipient cell to generate an engineered bacterium [68] pave the way for many applications previously thought to be out of reach [69]. The current understanding of bacterial genomic organization and its connection with precise phenotypic properties is insufficient to propose an optimized genome arrangement to the field of synthetic biology. MCH1 is by far the closest isogenic mono-chromosomal model that can be used to make comparisons with the bi-chromosomal *V. cholerae* N16961 strain. A previous work has been done in *Sinorhizobium meliloti*, in which spontaneous fusions of the three natural replicons occurs at low frequency through recombination between repeated sequences in the genome [70]. In these experiments, the three different fused molecules all conserved their functional origins of replication, and the resulting fusion was reversible, rendering the results inconclusive in terms of the relationship between growth advantage and genome organization. On the contrary, the single chromosome of our engineered MCH1 is stable, contains only a single origin and terminus of replication and therefore provides us with a powerful new tool to investigate the selective advantage(s) of the characteristic multipartite genome organization of vibrio*s*. New insights into bacterial genome organization and determination of how genomes are arranged can help us to design more optimized chromosomes, which will undoubtedly open novel developments in the field of synthetic biology.

## Methods

### Bacterial strains and growth conditions

Bacterial strains and plasmids used in this study are listed in Table S2. Cells were grown at 37°C in Luria broth. Antibiotics were used at the following concentrations: ampicillin, 75 µg/mL; chloramphenicol, 25 µg/mL for *E. coli* and 5 µg/mL for *V. cholerae*; kanamycin 25 µg/mL; spectinomycin 100 µg/mL; zeocin 25 µg/mL. Diaminopimelic acid was used at 0.3 mM, X-Gal (40 µg/mL); IPTG(1 mM); arabinose (0.2%) and glucose (1%).

### General cloning procedures

DNA cassettes containing the *att* recombination sites were transferred from a plasmid vector to the chromosome by two homologous recombination steps. To provide homology for integration, two 500 bp regions spanning the point of insertion were amplified from N16961 chromosomal DNA by PCR. The amplified fragments were cloned into an R6K γ-*ori*-based suicide vector, pSW7848 that encodes the *ccdB* toxin gene under the control of an arabinose-inducible and glucose-repressible promoter, *P_BAD_*. The sequences containing the *att* recombination sites of interest were then cloned between the two chromosomal fragments. For cloning, Π3813 was used as a plasmid host [71]. For conjugal transfer of plasmids to *V. cholerae* strains, *E. coli* β3914 was used as the donor [71]. Selection of the plasmid-borne drug marker resulted in integration of the entire plasmid in the chromosome by a single crossover. Elimination of the plasmid backbone resulting from a second recombination step was selected for by arabinose induction of the *ccdB* toxin gene.

### MCH1 construction

*V. cholerae* N16961 El Tor strain deleted for *lacZ* was used to create the mono-chromosomal MCH1 strain [4], [72]. Following the above-mentioned cloning and genome engineering procedures, four *attR/L* sites were inserted at precise chromosomal loci near *dif1* on chrI and near *oriCII* on chrII using pSW7848-derivitave KO vectors pMP36 (*attR_λ_*), pMP42 (*attL_λ_*), pMP35 (*attR_HK_*), pMP49 (*attL_HK_*) (Table S2). First, [*attR_λ_-3′lacZ-FRT-aph-FRT*] was inserted downstream of *rctB* on chrII using pMP36 in N16961Δ*lacZ* generating strain MV122. The *aph* cassette was excised using pCP20 for expression of Flp recombinase that catalyses recombination between the two *FRT* sites [73]–[74]. After Flp-mediated recombination, a single *FRT* site remained near *oriCII* and the strain became sensitive to kanamycin, MV122Δ*aph*. Second, [*attL_λ_-5′lacZ-FRT-aph-FRT*] was inserted upstream of *dif1* on chrI using pMP42 in MV122Δ*aph* generating strain MV124. The *aph* cassette was excised using pCP20, generating the mutant MV124Δ*aph*. We checked MV124Δ*aph* by PCR to make sure that undesirable recombination events between the remaining *FRT* site on chrII with *FRT* sites on chrI didn't occur. Third, *dif1* was replaced by [*attR_HK_-FRT-aph-FRT*] using pMP35, yielding strain MV125. To insert *attR_HK_* close to *dif1*, it was necessary to delete *dif1* to prevent site-specific integration of a *dif1*-carrying KO-vector mediated by the endogenous *V. cholerae* XerC/D recombinases [19]. The *aph* cassette was not excised, this antibiotic resistance cassette serving as a reporter to follow the subsequent loss of the excised 8 kb circular molecule resulting from the fusion of chr1 with chr2. Fourth, [*attL_HK_*] with no antibiotic resistance cassette was inserted downstream of *parB2* using pMP49 generating mutant MV127 (Figure 1A).

A temperature-sensitive replicating vector pMP6 expressing [*int_λ_-xis_λ_ , int_HK_-xb is_HK_*] was conjugated into MV127. Donor cells β2163 [pMP6] and recipient cells (MV127) were conjugated for one hour at 30°C and plated on LB-agar at 30°C supplemented with ampicillin, X-Gal and IPTG to select for pMP6 and monitor recombination events between *attL_λ_* and *attR_λ_*. Reconstitution and expression of the β-galactosidase encoding gene led to appearance of blue cells when grown in presence of X-Gal and IPTG. After 36 hours of growth at 30°C, blue quarters appeared within single white conjugant colonies (Figure 1E). From blue/white colonies of mixed population, cells were grown at 30°C in LB in presence of ampicillin to enrich for chromosome rearrangements. Cells were plated on LB supplemented with X-Gal, IPTG to monitor *attL_λ_* and *attR_λ_* recombination events and incubated at 42°C to cure pMP6. All selected colonies were completely blue. Ten clones were isolated and tested by PCR using primers flanking both recombined *attB_λ_* and *attB_HK_* sites to verify that recombination occurred between all four recombination sites. All tested blue clones also had recombined *attR_HK_*×*attL_HK_*. Fusion of the two chromosomes resulted in the excision of an 8 kb circular molecule. In absence of antibiotic pressure that selected for this 8 kb circular molecule (*aph* gene formerly located in the terminus region of chrI), the molecule was rapidly lost. All remaining and undesired *FRT* and *attP* sites were excised within the 8 kb molecule and subsequently lost. The resulting mutant carries a single circular chromosome, free of antibiotic resistance cassettes and containing only two short 50 bp *attB* sites that delimit chrI from chrII. Genomic stability of the mutant was established over 1000 generations carried out during a long-term evolution experiment.

### Pulsed field gel electrophoresis

The preparation of genomic DNA embedded in agarose gels and the protocol for PFGE was performed as previously described [8], [75].

### Dam depletion

WT, MCH1 and ICO1 strains were deleted for *dam* using pGD121 knock-out vector in the presence of pGD93 (Dam complementing vector) and then depleted for Dam as previously described [9].

### Growth competition assay

The proportion of cells that a mutant strain deficient in dimer resolution fails to produce at each doubling time of its parent can be measured by growth competition experiments. Growth competitions of *V. cholerae* strains are described in [19]. *V. cholerae* cells were grown at 37°C with a 10^−3^ dilution in LB media every 8–12 h. Colony-forming units (CFUs) of mutant and parental cells in the cultures were determined by plating on appropriate antibiotic plates. These numbers were used to calculate the number of generations of the parent cells between each time points and the CFUs ratio of mutant versus parent cells at each time point. This ratio varies exponentially with the number of generations. The coefficient of this exponential is a good estimation of the rate of dimer formation [19]. Following this method, we estimated the rate of dimer formation for each mutant in three independent experiments. In Figure 3C, the relationship between the rate of dimer formation and the logarithm of chromosome size has a very high R^2^ (>0.9) with no significant departure from linearity (P value = 0.1827), which indicates a strong linear relationship between the two variables. The slope is significantly different from zero (P value<0.0001) and the confidence interval for the slope is 95%.

## Supporting Information

Figure S1 MCH1 has a replication pattern consistent with a single chromosome replicating at constant rate. The Cooper-Helmstetter model for DNA replication [76] predicts the DNA distribution in an ideal culture and replication parameters can be estimated from computer-simulations of the DNA histograms [16], [77]–[78]. Cultures of *V. cholerae*, WT (left panels) or MCH1 (right panels), were grown exponentially with different carbon-sources to obtain independent samples with different cell-cycle parameters and samples were analyzed by flow cytometry. We compared the experimental DNA histograms obtained by flow cytometry to computer simulations of DNA contents in ideal cultures using the approach described by Michelsen *et al* [77]. The DNA histograms were simulated assuming either two chromosomes (WT) or one chromosome (MCH1) as described by [16]. In these simulations, the DNA histograms are resolved into the contributions from cells in the B, C and D periods. Shown are samples grown in M9+fructose (upper panels) and M9+fructose+serine (lower panels). Purple dots are actual DNA contents data, green curves simulate pre-replicating (B period) cells, blue curves simulate replicating (C period) cells, red curves simulate post-replicating (D period) cells and black curves accumulates the B, C and D period cells. The difference between the one and two chromosome simulations shows mainly in the shape of distribution of replicating cells: the increased replication rate late in the cell cycle with both chromosomes replicating lowers the blue C-curve compared to the same curved in cells with one chromosome.
(TIF)

Figure S2 Construction of a mutant of *V. cholerae*, ESC1, with equally sized chromosomes. A. *V. cholerae* MV155 strain with *attR/L* sites from λ and HK022 phages inserted at precise loci. Recombination sites are located as follows: *attR_HK_* in the intergenic region of [VC1939–VC1940] and *attL_λ_* in the intergenic region of [VC981–VC982] on chrI; *attL_HK_* in the intergenic region of [VCA628–VCA629] and *attR_λ_* in the intergenic region of [VCA514–VCA515] on chrII. B. Recombination [*attR_λ_*×*attL_λ_*] and [*attR_HK_*×*attL_HK_*] mediated by the expression of Int_λ_+Xis_λ_ and Int_HK_+Xis_HK_. C. Recombination events [*attR_λ_*×*attL_λ_*] regenerate *lacZ*, allowing for phenotypic detection of rearranged chromosomes. Recombination [*attR_λ_*×*attL_λ_*] and [*attR_HK_*×*attL_HK_*] leads to the transfer of 1 Mbp from chrI to chrII and the exchange of *dif1* and *dif2* sites. D. Ethidium bromide stained pulse-field-gel electrophoresis of genomic DNA: lane 1, WT; lane 2, MCH1; lane 3, ESC1; lane 4, *H. wingei* marker (BioRad).
(TIF)

Table S1 Generation time of various genomic mutants in fast growing conditions.
(DOC)

Table S2 List of plasmids and bacterial strains.
(DOC)

Text S1 Supporting methods.
(DOCX)
