# Supplementary figures and images for: Genome Engineering in Vibrio cholerae: A Feasible Approach to Address Biological Issues
Source: PLoS Genet. 2012 Jan 12;8(1):e1002472. doi: 10.1371/journal.pgen.1002472 (PMC3257285; doi:10.1371/journal.pgen.1002472)

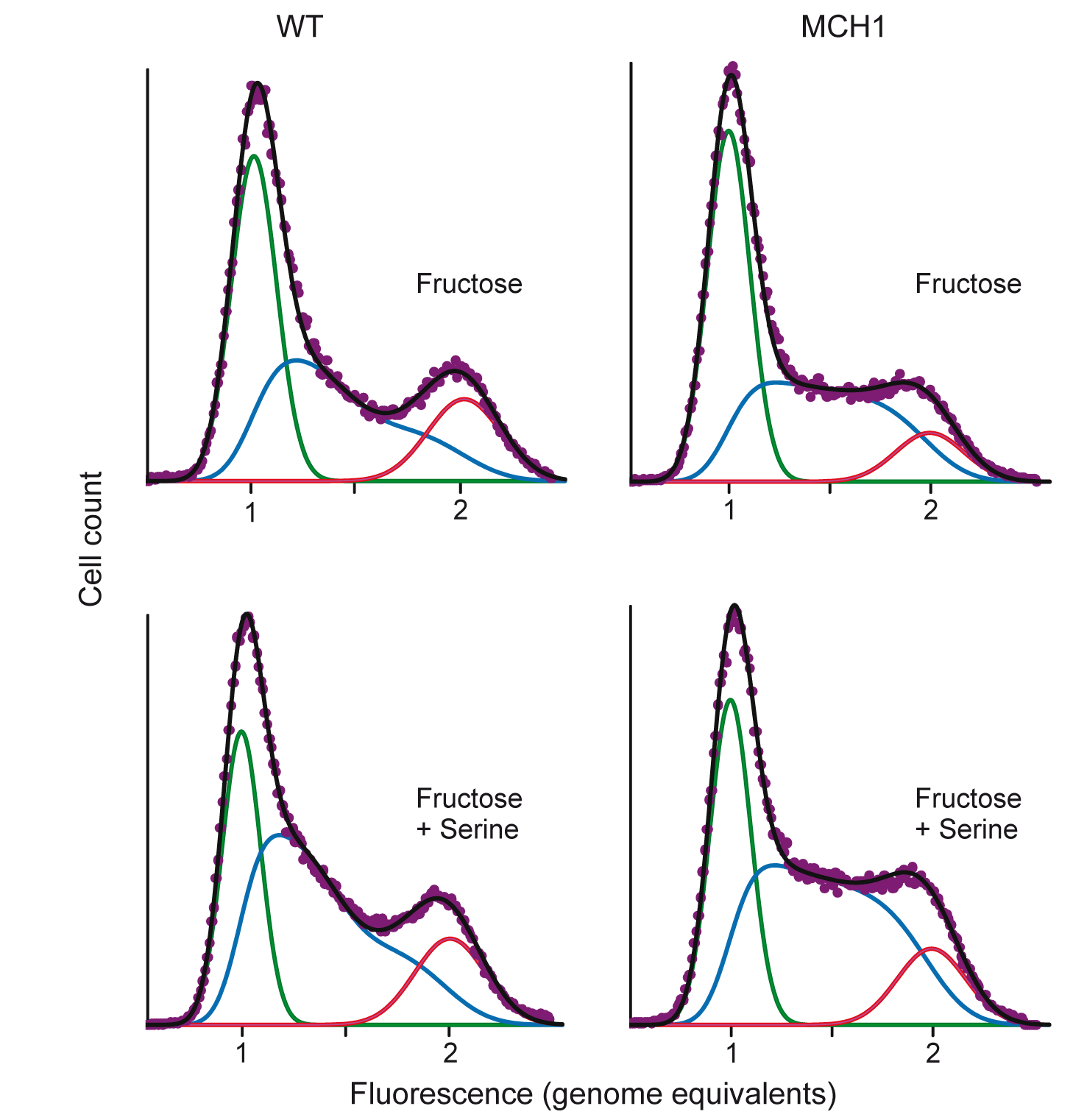

Supplement: Figure S1 — MCH1 has a replication pattern consistent with a single chromosome replicating at constant rate. The Cooper-Helmstetter model for DNA replication [76] predicts the DNA distribution in an ideal culture and replication parameters can be estimated from computer-simulations of the DNA histograms [16], [77]–[78]. Cultures of V. cholerae, WT (left panels) or MCH1 (right panels), were grown exponentially with different carbon-sources to obtain independent samples with different cell-cycle parameters and samples were analyzed by flow cytometry. We compared the experimental DNA histograms obtained by flow cytometry to computer simulations of DNA contents in ideal cultures using the approach described by Michelsen et al [77]. The DNA histograms were simulated assuming either two chromosomes (WT) or one chromosome (MCH1) as described by [16]. In these simulations, the DNA histograms are resolved into the contributions from cells in the B, C and D periods. Shown are samples grown in M9+fructose (upper panels) and M9+fructose+serine (lower panels). Purple dots are actual DNA contents data, green curves simulate pre-replicating (B period) cells, blue curves simulate replicating (C period) cells, red curves simulate post-replicating (D period) cells and black curves accumulates the B, C and D period cells. The difference between the one and two chromosome simulations shows mainly in the shape of distribution of replicating cells: the increased replication rate late in the cell cycle with both chromosomes replicating lowers the blue C-curve compared to the same curved in cells with one chromosome. (TIF) [file pgen.1002472.s001.tif]

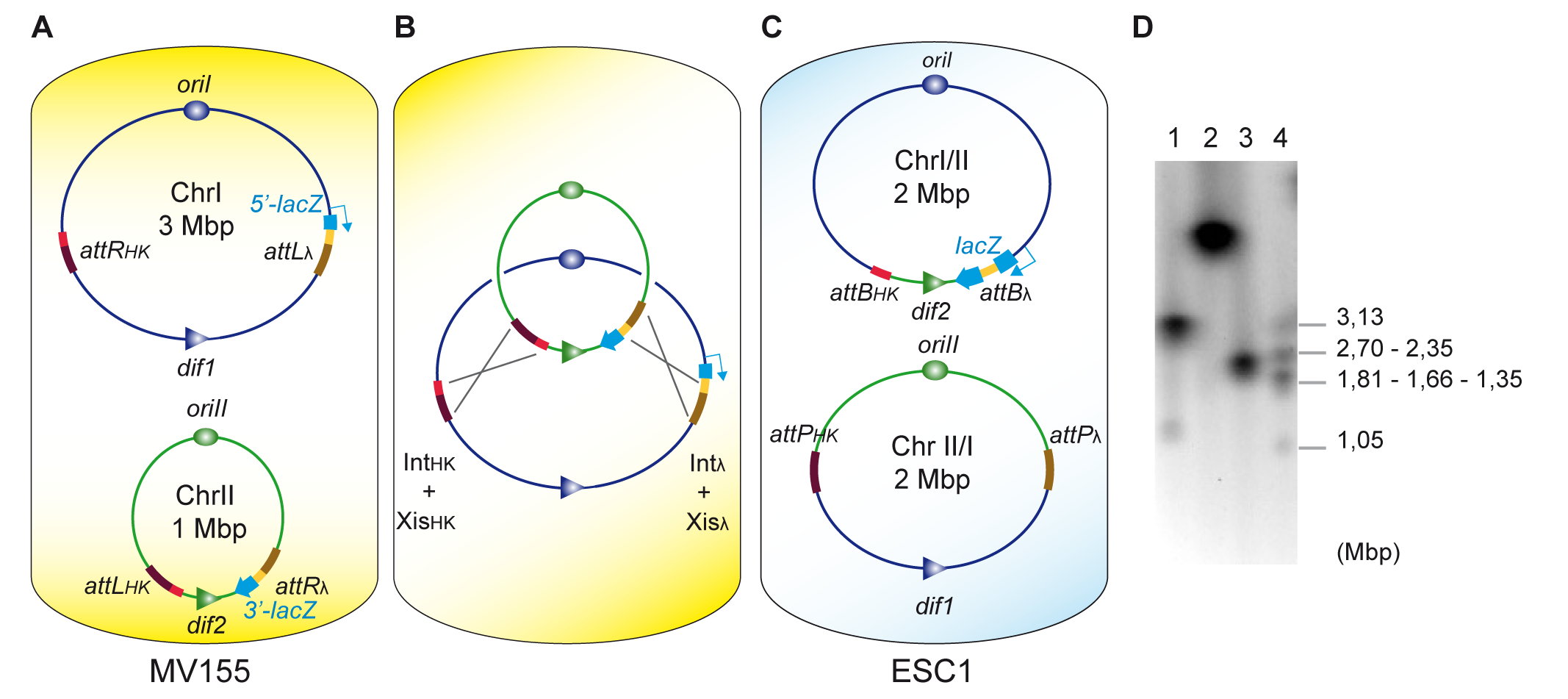

Supplement: Figure S2 — Construction of a mutant of V. cholerae, ESC1, with equally sized chromosomes. A. V. cholerae MV155 strain with attR/L sites from λ and HK022 phages inserted at precise loci. Recombination sites are located as follows: attRHK in the intergenic region of [VC1939–VC1940] and attLλ in the intergenic region of [VC981–VC982] on chrI; attLHK in the intergenic region of [VCA628–VCA629] and attRλ in the intergenic region of [VCA514–VCA515] on chrII. B. Recombination [attRλ×attLλ] and [attRHK×attLHK] mediated by the expression of Intλ+Xisλ and IntHK+XisHK. C. Recombination events [attRλ×attLλ] regenerate lacZ, allowing for phenotypic detection of rearranged chromosomes. Recombination [attRλ×attLλ] and [attRHK×attLHK] leads to the transfer of 1 Mbp from chrI to chrII and the exchange of dif1 and dif2 sites. D. Ethidium bromide stained pulse-field-gel electrophoresis of genomic DNA: lane 1, WT; lane 2, MCH1; lane 3, ESC1; lane 4, H. wingei marker (BioRad). (TIF) [file pgen.1002472.s002.tif]
