# Supplementary material for: Genome Engineering in Vibrio cholerae: A Feasible Approach to Address Biological Issues
Source: PLoS Genet. 2012 Jan 12;8(1):e1002472. doi: 10.1371/journal.pgen.1002472 (PMC3257285; doi:10.1371/journal.pgen.1002472)
Supplement: Table S1 — Generation time of various genomic mutants in fast growing conditions. (DOC) [file pgen.1002472.s003.doc]

**Table S1. Generation time of various genomic mutants in fast growing conditions.**

| **Strain** | **Generation Time*** |
| --- | --- |
| WT | 23 (+/- 1.3) |
| MCH1 | 29 (+/- 0.3) |
| ICO1 | 31 (+/- 1.8) |
| ESC1 | 28 (+/- 0.3) |

* Generation time average values in minutes (+/- standard deviation) of exponentially growing cells in rich medium (AB minimal medium [5] supplemented with 0.2% glucose and 1% casamino acids).
