# Supplementary material for: Genome Engineering in Vibrio cholerae: A Feasible Approach to Address Biological Issues
Source: PLoS Genet. 2012 Jan 12;8(1):e1002472. doi: 10.1371/journal.pgen.1002472 (PMC3257285; doi:10.1371/journal.pgen.1002472)
Supplement: Table S2 — List of plasmids and bacterial strains. (DOC) [file pgen.1002472.s004.doc]

**Table S2. List of plasmids and bacterial strains**

| **Name** | **Relevant genotype or features** | **Reference** |
| --- | --- | --- |
| **Plasmids** | | |
| pSW7848 | Suicide plasmid for allele exchange - *oriV*_R6Kγ_ *oriT*_RP4_ *araC*-P_BAD_-*ccdB* | This study |
| pMP36 | pSW7848 [*attR_λ_*-3' *lacZ*-*FRT*-*aph*-*FRT*] cassette flanked by 2 homologous regions downstream of *oriII*-*rctB* (chrII) | This study |
| pMP42 | pSW7848 [*attL_λ_*-5' *lacZ*-*FRT*-*aph*-*FRT*] cassette flanked by 2 homologous regions upstream of *dif1* (chrI) | This study |
| pMP35 | pSW7848 Δ*dif1*::[*attR_HK_*-*FRT*-*aph*-*FRT*] cassette flanked by 2 homologous regions surrounding *dif1* (chrI) | This study |
| pMP49 | pSW7848 [*attL_HK_*] cassette flanked by 2 homologous regions downstream of *parB2* (chrII) | This study |
| pMP89 | pSW7848 [*attL_HK_*-*FRT*-*cat*-*FRT*] cassette flanked by 2 homologous regions upstream of *oriII*-*rctB* (chrII) | This study |
| pMP50 | pSW7848 [*attR_HK_*-*oriCI*-*FRT*-*arr2*-*FRT*- *attL_λ_*-5' *lacZ*] | This study |
| pCP20 | pSC101*rep*(Ts) [*flp*] | [6] |
| pMEV250 | pSC101 [*oriT*_RP4_ *araC*-P_BAD_-*cre*] | This study |
| pMP6 | pSC101*rep*(Ts) *oriT*_RP4_ [*int_λ_-xis_λ_, int_HK_-xis_HK_*] | This study |
| pGD93 | pSC101*rep*(Ts) P_BAD-_*dam* | [7] |
| pGD121 | pDS132 Δ*dam*::*zeo* | [7] |
| ***E. coli*** | | |
| Π1 | DH5α Δ*thyA*::(*erm*-*pir*116) | [8] |
| β2163 | (F-) RP4-2-Tc::Mu Δ*dapA*::(*erm*-*pir*116) | [8] |
| Π3813 | B462 Δ*thyA*::(*erm*-*pir*116) | [9] |
| β3914 | β2163 *gyr*A462 *zei*-298::Tn10 | [9] |
| ***V.cholerae*** | | |
| N16961 | *Vibrio cholerae* serotype O1 biotype El Tor strain N16961 | [10] |
| N16961*ΔlacZ* | N16961 Δ*lacZ* using pMEV69 | This study |
| MV122 | N16961 Δ*lacZ*, [*attR_λ_*-3' *lacZ*-*FRT*-*aph*-*FRT*] (between *rctB* and VCA003) using pMP36 | This study |
| MV122Δ*aph* | MV122 Δ*aph* using pCP20 (excisive recombination at *FRT* sites) | This study |
| MV124 | MV122 Δ*aph*, [*att*L_λ_-5' *lac*Z-*FRT*-*aph*-*FRT*] (between *rtxA* and *dif1*) using pMP42 | This study |
| MV124Δ*aph* | MV124 Δ*aph* using pCP20 (excisive recombination at *FRT* sites) | This study |
| MV125 | MV124 Δ*aph*, Δ*dif1*::[*attR_HK_*-*FRT*-*aph*-*FRT*] using pMP35 | This study |
| MV127 | MV125 Δ*lac*Z, [*attL_HK_*] (between VCA1113 and *parB2*) using pMP49 | This study |
| MCH1 | Fusion of chrI and chrII in MV127 using pMP6 | This study |
| MV140 | MV122, [*attL_HK_*-*FRT*-*cat*-*FRT*] (between *rctA* and *rctB*) using pMP89 | This study |
| ICO1 | chrII substitution with chrI of MV140 using pMP50 and pMP6 | This study |
| N16961*ChapR* | N16961::mTn*7hapR* | [1] |
| N16961*ChapRΔlacZ* | N16961C*hap*R Δ*lacZ* using pMEV69 | This study |
| MV149 | N16961C*hap*RΔ*lac*Z, [*attL_HK_*-*FRT*-*aph*-*FRT*] (between VCA628 and VCA629) | This study |
| MV151 | MV149, [*attR_λ_*-3' *lacZ*-*loxP*-*cat*-*loxP*] (between VCA514 and VCA515) | This study |
| MV151Δ*aph* | Deletion of *aph* in MV151 using pCP20 (excisive recombination at *FRT* sites with Flp) | This study |
| MV151Δ*aph*Δ*cat* | Deletion of *cat* in MV151Δ*aph* using pMEV250 (excisive recombination at *loxP* sites with Cre) | This study |
| MV154 | MV151Δ*aph*Δ*cat*, [*attL_λ_*-5' *lacZ*-*FRT*-*aph*-*FRT*] (between VC981 and VC982) | This study |
| MV155 | MV154, [*attR_HK_*-*FRT*-*cat*-*FRT*] (between VC1939 and VC1940) | This study |
| ESC1 | chrI and chrII recombinational exchange in MV155 using pMP6 | This study |
| N16961 Δ*dam* / pGD93 | N16961 Δ*dam* using pGD121 in presence of pGD93 | This study |
| MCH1 Δ*dam* / pGD93 | MCH1 Δ*dam* using pGD121 in presence of pGD93 | This study |
| MCH1 Δ*dam* | MCH1 Δ*dam* (after loss of *dam*-complementing plasmid pGD93) | This study |
| ICO1 Δ*dam* / pGD93 | ICO1 Δ*dam* using pGD121 in presence of pGD93 | This study |
| ICO1 Δ*dam* | ICO1 Δ*dam* (after loss of *dam*-complementing plasmid pGD93) | This study |
| WTΔ*dif1* | N16961C*hap*R Δ*lacZ* Δ*dif1::aadA7* | This study |
| WTΔ*dif2* | N16961C*hap*R Δ*lacZ* Δ*dif2*::*aadA7* | This study |
| WTΔ*xerC* | N16961C*hap*R Δ*lacZ* Δ*xerC*::*aadA7* | This study |
| MCH1*ChapR* | MCH1::mTn7*hapR* | This study |
| MCH1Δ*dif2* | MCH1*ChapR* Δ*dif1*::*aadA7* | This study |
| ESC1Δ*dif1* | ESC1 Δ*dif1*::*aadA7* | This study |
| ESC1Δ*dif2* | ESC1 Δ*dif2*::*aadA7* | This study |
| ESC1Δ*xerC* | ESC1 Δ*xerC*::*aadA7* | This study |
