# Supplementary material for: Genome Engineering in Vibrio cholerae: A Feasible Approach to Address Biological Issues
Source: PLoS Genet. 2012 Jan 12;8(1):e1002472. doi: 10.1371/journal.pgen.1002472 (PMC3257285; doi:10.1371/journal.pgen.1002472)
Supplement: Text S1 — Supporting methods. (DOCX) [file pgen.1002472.s005.docx]

**Text S1**

**ICO1 construction**

Following the previously described cloning and genome engineering procedures, two *att* sites were inserted on chrII adjoining *oriII*: *attR_λ_* was inserted between *rctB* and VCA003 in N16961Δ*lac*Z giving strain MV122; *attL_HK_* was inserted between *rctA* and *oriII* in MV122 giving strain MV140 (Table S2). A conjugative suicide vector, pMP50, carrying a cassette with *oriI* adjacent to *arr2* (conferring resistance to rifampicin) flanked by *attR_HK_* and *attL_λ_* and encoding *ccdB* toxin gene on its backbone was conjugated into MV140. First, we observed that pMP50 was able to replicate autonomously, demonstrating the ARS activity of the *oriI*-containing cassette and that chromosomal and extrachromosomal copies of *oriI* can “cohabit” in *V. cholerae*. Secondly, we introduced pMP6 expressing [*int_λ_-xis_λ_, int_HK_-xis_HK_*]. Recombination between chrII and the suicide vector led to the replacement of [*oriII-rctB*] from chrII with *oriI* from the vector. The complete recombination product was selected using a positive selection marker (*arr2*) and a negative selection marker (*ccdB*). The resulting mutant ICO1 carries two identical *oriI* chromosomal origins.

**ESC1 construction**

MCH1 and ICO1 were constructed using traditional methods, which required several DNA cloning steps into a suicide vector in *E. coli* and a subsequent conjugational transfer into *V. cholerae.* The last mutant ESC1 was constructed differently, using a simpler mutagenesis method that was more recently developed in *V. cholerae*. This technique is based on *V. cholerae's* ability to acquire natural competence upon growth on chitin surfaces [1]. Natural competence enables bacteria to take up free DNA from the environment in order to incorporate it into their genome [1]. *V. cholerae* N16961 is not naturally transformable because of a frameshift mutation in the hapR gene, the quorum-sensing regulator [1]. To create ESC1, we used *V. cholerae* N16961 that has been complemented with a wild-type copy of hapR from the transformable strain A1552 [1]. Consequently we complemented WT, MCH1 and ICO1 with a functional copy of hapR to be able to compare them with ESC1 for further studies.

Genome alteration of *V. cholerae* involved homologous recombination between the chromosomal region of interest and a PCR product generated by two-step PCR that contains an antibiotic resistance cassette flanked by at least 500 bp sequences homologous to the target DNA. Following the procedure described in [2], four *attR/L* sites were inserted in N16961C*hap*RΔ*lac*Z at precise chromosomal loci (Table S2). Chronologically, *att*L_HK_ was inserted between VCA628 and VCA629 in N16961C*hap*RΔ*lac*Z giving strain MV149; *att*R_λ_ was inserted between VCA514 and VCA515 in MV149 giving strain MV151; antibiotic markers were excised giving MV151Δ*aph*Δ*cat*; *att*L_λ_ was inserted between VC981 and VC982 in MV151Δ*aph*Δ*cat* giving strain MV154 and finally *att*R_HK_ was inserted between VC1939 and VC1940 in MV154 giving strain MV155 (Figure S2A). The vector pMP6 expressing [*int_λ_-xis_λ_, int_HK_-xis_HK_*] was conjugated into MV155 to allow for the *att*R/L sites to recombine with each other (Figure S2B). Selection of the chromosome rearranged mutant was carried out as previously described for MCH1. The resulting mutant ESC1 carries two equally sized chromosomes of approximately 2 Mbp (Figure S2C). ESC1 was confirmed by PFGE, the two chromosomes of equal size overlap giving one visible band at around 2 Mbp (Figure S2D).

**Microscopy**

Fixed cells: cells were grown until OD_450_ ~ 0.2 and treated with chloramphenicol (20 μg/mL) for 3 hours under agitation at 37°C. Incubation in chloramphenicol causes nucleoids to condense, and connections between nucleoids are easily seen [3]. Cells were fixed with 5% paraformaldehyde, 0.06% glutaraldehyde in 1X PBS, stained with DAPI (2 μg/mL) for 20 min at 4°C on a rotating wheel and washed two times with 1X PBS. Fixed cells were settled on a microscope slide coated with a solid matrix of 1% agarose (in 1X PBS). Microscopy was carried out on a [Zeiss Axiovert 200M](http://www.zeiss.de/axiovert200) inverted fluorescence microscope, equipped with a [Photometrics Cool snap HQ](http://www.photomet.com/coolsnap.html#hq) camera. Images were acquired using Axiovision software (Zeiss).

**Flow cytometry and cell-cycle simulations**

Flow cytometry was performed as already described [4] using an Apogee A10 instrument (Apogee Flow Systems Inc.). The DNA distributions in ideal cultures were simulated using a custom Excel worksheet that accommodate for the presence of two chromosomes as already described [5].

**Supplementary references**

1. Meibom KL, Blokesch M, Dolganov NA, Wu CY, Schoolnik GK (2005) Chitin induces natural competence in *Vibrio cholerae*. Science 310: 1824-1827.

2. Marvig RL, Blokesch M (2010) Natural transformation of *Vibrio cholerae* as a tool--optimizing the procedure. BMC Microbiol 10: 155.

3. Steiner WW, Kuempel PL (1998) Cell division is required for resolution of dimer chromosomes at the *dif* locus of *Escherichia coli*. Mol Microbiol 27: 257-268.

4. Lobner-Olesen A, Skarstad K, Hansen FG, von Meyenburg K, Boye E (1989) The DnaA protein determines the initiation mass of *Escherichia coli* K-12. Cell 57: 881-889.

5. Rasmussen T, Jensen RB, Skovgaard O (2007) The two chromosomes of *Vibrio cholerae* are initiated at different time points in the cell cycle. EMBO J 26: 3124-3131.

6. Cherepanov PP, Wackernagel W (1995) Gene disruption in *Escherichia coli*: TcR and KmR cassettes with the option of Flp-catalyzed excision of the antibiotic-resistance determinant. Gene 158: 9-14.

7. Demarre G, Chattoraj DK (2010) DNA adenine methylation is required to replicate both *Vibrio cholerae* chromosomes once per cell cycle. PLoS Genet 6: e1000939.

8. Demarre G, Guerout AM, Matsumoto-Mashimo C, Rowe-Magnus DA, Marliere P, et al. (2005) A new family of mobilizable suicide plasmids based on broad host range *R388* plasmid (*IncW*) and *RP4* plasmid (*IncPalpha*) conjugative machineries and their cognate *Escherichia coli* host strains. Res Microbiol 156: 245-255.

9. Le Roux F, Binesse J, Saulnier D, Mazel D (2007) Construction of a *Vibrio splendidus* mutant lacking the metalloprotease gene *vsm* by use of a novel counterselectable suicide vector. Appl Environ Microbiol 73: 777-784.

10. Heidelberg JF, Eisen JA, Nelson WC, Clayton RA, Gwinn ML, et al. (2000) DNA sequence of both chromosomes of the cholera pathogen *Vibrio cholerae*. Nature 406: 477-483.
